# Bioguided Isolation of Active Compounds from *Rhamnus alaternus* against Methicillin-Resistant *Staphylococcus aureus* (MRSA) and Panton-Valentine Leucocidin Positive Strains (MSSA-PVL)

**DOI:** 10.3390/molecules26144352

**Published:** 2021-07-19

**Authors:** Ikrame Zeouk, Wessal Ouedrhiri, Ines Sifaoui, Isabel L. Bazzocchi, José E. Piñero, Ignacio A. Jiménez, Jacob Lorenzo-Morales, Khadija Bekhti

**Affiliations:** 1 Instituto Universitario De Enfermedades Tropicales y Salud Pública de Canarias, Universidad de la Laguna, Avda. Astrofísico Fco. Sánchez, S/N, 38203 La Laguna, Spain; isifaoui@ull.edu.es; 2 Laboratory of Microbial Biotechnology and Bioactive Molecules, Department of Biology, Faculty of Sciences and Techniques, Sidi Mohamed Ben Abdellah University, Fez 2202, Morocco; bekhti.bki4@gmail.com; 3 Laboratory of Engineering, Electrochemistry, Modeling and Environment, Department of Chemistry, Faculty of Science, Sidi Mohamed Ben Abdellah University, Fez 2202, Morocco; wessal.ouedrhiri@gmail.com; 4 Departamento de Obstetricia, Ginecología, Pediatría, Medicina Preventiva y Salud Pública, Toxicología, Medicina Legal y Forense y Parasitología, Universidad de la Laguna, 38203 La Laguna, Spain; 5 Red de Investigación Colaborativa en Enfermedades Tropicales (RICET), Instituto de Salud Carlos III, 28029 Madrid, Spain; 6 Departamento de Química Orgánica, Instituto Universitario de Bio-Orgánica Antonio González, Universidad de La Laguna, Avenida Astrofísico Francisco Sánchez 2, 38206 La Laguna, Spain; ilopez@ull.edu.es (I.L.B.); ignadiaz@ull.edu.es (I.A.J.)

**Keywords:** ethnobotany, bioguided fractionation, MRSA, MSSA-PVL, anti-staphylococcal activity, *Rhamnus alaternus*, emodin

## Abstract

Despite intensified efforts to develop an effective antibiotic, *S. aureus* is still a major cause of mortality and morbidity worldwide. The multidrug resistance of bacteria has considerably increased the difficulties of scientific research and the concomitant emergence of resistance is to be expected. In this study we have investigated the in vitro activity of 15 ethanol extracts prepared from Moroccan medicinal plants traditionally used for treatment of skin infections. Among the tested species *I. viscosa*, *C. oxyacantha*, *R. tinctorum*, *A. herba alba,* and *B. hispanica* showed moderate anti-staphylococcal activity. However, *R. alaternus* showed promising growth-inhibitory effects against specific pathogenic bacteria especially methicillin-susceptible *Staphylococcus aureus* Panton-Valentine leucocidin positive (MSSA-PVL) and methicillin-resistant *S. aureus* (MRSA). The bioguided fractionation of this plant using successive chromatographic separations followed by nuclear magnetic resonance (NMR) and mass spectrometry (MS) including EIMS and HREIMS analysis yielded the emodin (**1**) and kaempferol (**2**). Emodin being the most active with MICs ranging between 15.62 and 1.95 µg/mL and showing higher activity against the tested strains in comparison with the crude extract, its mechanism of action and the structure-activity relationship were interestingly discussed. The active compound has not displayed toxicity toward murine macrophage cells. The results obtained in the current study support the traditional uses of *R. alaternus* and suggest that this species could be a good source for the development of new anti-staphylococcal agents.

## 1. Introduction

According to the WHO, 50,000 persons around the world are dying every day because of infectious diseases. In the World Health Report, more than 17 million deaths out of a total of 52 million were due to infectious diseases [1]. Among many of these disorders, *Staphylococcus aureus* (*S. aureus*) is the main responsible pathogenic bacteria with a large clinical spectrum [2]. Within this species there are toxigenic variants such as those expressing Panton-Valentine Leucocidin (PVL) or those expressing Staphylococcal Toxic Shock Toxin (STST). Indeed, PVL is known worldwide for its potential role in virulence and for its involvement in invasive infections. Although only 5% of *S. aureus* strains produce PVL (Sa-PVL), it is a widely studied toxin. Currently, Duployez et al. [3] have reported a fatal case of a young adult with COVID-19; the complication of viral infection has been linked to necrotizing pneumonia induced by *S. aureus* producing PVL. Therefore, these toxigenic strains make *S. aureus* infections more dangerous requiring specific clinical treatment [4,5]. However, despite the abundance of numerous classes of antibiotics, the emergence of resistant strains is increasing. *S. aureus* has developed multi-drug resistance toward different antibiotics, especially methicillin (MRSA). MRSA strains include healthcare-associated MRSA (HA-MRSA) and community-associated MRSA (CA-MRSA) aspects which may be an increased risk of spreading the *S. aureus* infections [6] ranging from skin to other invasive diseases such as pneumonia [7], meningitis [8], sepsis [9], osteomyelitis [10], and infective endocarditis [11]. The newest antibiotic on the market (linezolid (Zyvox)) was introduced to the North American market in 2000 [12]. Since 2001, cases of linezolid-resistant MRSA were reported [13,14]. To this end, it becomes necessary to search for new safe and efficient anti-staphylococcal agents. Higher plants have been widely described as a rich library for the identification of new active compounds. *Rhamnus alaternus* belonging to the Rhamnaceae family is a promising resource of phytochemicals, it is a perennial dioecious shrub and an important species of the Mediterranean area widely used in folk medicine [15]. Previous phytochemical analysis has shown that the *Rhamnus* genus is rich in flavonols such as kaempferol, rhamnetin, and rhamnocitrin [16]. Other investigations have revealed the presence of anthocyanins in *R. alaternus* berries in addition to anthraquinones such as emodin [17,18,19]. Furthermore, several studies have already explored the biological activities of this plant highlighting numerous pharmacological properties such as antihyperlipidemic, antigenotoxic, and antioxidant effects [20,21,22]. However, to the best of our knowledge, no in-depth bioguided fractionation of this plant has been conducted against pathogenic bacteria and especially MRSA and Sa-PVL. In the present work, we describe a bioguided fractionation of *R. alaternus* ethanolic extract leading to the isolation and characterization of anthraquinones and flavonoids, we also report the compound which is most likely be involved in the antibacterial activity.

## 2. Materials and Methods

### 2.1. General Procedure

All solvents used were of analytical grade from Sigma-Aldrich. Silicagel 60 F_254_ (Merck -Darmstadt, Germany) plates (20 × 20 cm) supported on aluminum sheet were used for thin-layer chromatography (TLC) separations. Silicagel 60 (Fluka-chemie GmbH) for column chromatography was obtained from Sigma-Aldrich. For the purification procedure, Silica gel 60 (particle size 15–40 and 63–200 μm, Macherey-Nagel) was used for column chromatography (CC), while silica gel 60 F254 was used for analytical TLC. The developed TLC plates were visualized by UV light and then spraying with HOAc-H_2_SO_4_-H_2_O (80:16:4) system, followed by heating at 100 °C during 3 min. ^1^H NMR spectrum was carried out on a Bruker Avance 600 spectrometer, with the pulse sequences given by Bruker. The electron impact mass spectrometry (EIMS) and high-resolution electron ionization mass spectrometry (HREIMS) were measured on an LCT Premier XE Micromass Electrospray Spectrometer.

### 2.2. Plant Material and Extraction

Plants were collected from the Atlas Mountains of Imouzzer region-Morocco in July, 2017 and were identified by Pr EL OUALIDI. J. and Pr IBN TATTOU. M. Each plant part was extracted by 6 h maceration with ethanol (96%) (1:10 *w/v*) at room temperature with continuous stirring at 500 rpm and then air-dried and ground to a powder. A voucher specimen of the active species was conserved under the reference number “RAB107343” in the Herbarium of the Scientific Institute, Rabat, Morocco.

### 2.3. Bacterial Strains

There are a multitude of *Staphylococcus aureus* strains, each one has specific characteristics. To this end, it is necessary to link each selected strain to specific effects. The strains selected for the present study are characterized by several properties, considering the community of origin, the antibiogram profile, and the absence or presence of PVL toxin, therefore, four strains were selected: three clinical isolates: *S. aureus* (*Sa*), *S. aureus* (MRSA348), *S. aureus* PVL (*Sa*PVL+), and a methicillin-resistant reference strain (MRSA (ATCC29213)). All strains with a verified antibiogram profile (Tables S1 and S2) are resistant to methicillin except for the toxigenic strain (*Sa*PVL+) which is sensitive. The antibiogram profile of the studied strains was identified at the Laboratory of Bacteriology in Fez-Morocco. In addition, these strains are often involved in cutaneous infections and other invasive infectious diseases.

### 2.4. Antibacterial Testing

In the present work, the evaluation of anti-staphylococcal activity was carried out by a combination of diffusion and dilution methods. After evaluating of the sensitivity of the strains toward DMSO, the fifteen ethanolic extracts that were previously dissolved at a concentration of 50 mg/mL in 2% (*v/v*) DMSO were screened against the four strains of *S. aureus* previously described.

#### 2.4.1. Inoculum’s Preparation

Revivification of bacteria was performed by subculturing the agar plate surface Luria-Bertani (LB) and incubated at 37 °C for 18 to 24 h. The microbial inoculums were obtained from fresh colonies through the direct colony suspension method. Hence, 1 to 2 colonies were suspended in a sterile saline solution (NaCl 0.9%) and adjusted to 0.5 McFarland scale (10^8^ CFU/mL).

#### 2.4.2. Agar Well Diffusion Assay

The preliminary screening of different extracts was carried out using the well diffusion method. The agar surface was inoculated by spreading 1 mL of the bacterial suspension. A vertical hole of 6 mm was punched aseptically and a volume of 80 to 100 μL of each extract at 50 mg/mL was introduced into each well. Finally, the plates were incubated for 24 h at 37 °C.

#### 2.4.3. Agar Dilution Assay

Since the tested extracts are colored, they may mask the detection of the microbial growth in the liquid medium. For this reason, the agar dilution method was performed for the MIC determination as described by Balouiri et al. [23] with slight modifications. This method consists of incorporating the extract at varying concentrations into the agar medium before its solidification (50 °C). Thus, different concentrations of the crude extracts were prepared in diluted DMSO, then 1 mL of each dilution was incorporated into 9 mL of sterile LB to get a serial two-fold dilutions ranging from 16 to 0.5 mg/mL. The obtained mixture was well shaken and distributed into Petri dishes. After the medium solidification, the agar surface was inoculated by spots of 5 µL of the inoculum of 10^5^ CFU/mL. The plates were incubated for 24 h at 37 °C. A control was performed without extract.

### 2.5. Bioguided Fractionation and Purification of Bioactive Compounds from the Leaves Extract of R. alaternus

#### 2.5.1. Bioautography

Using different mixtures of organic solvents, a preliminary study of polarity was performed to select the optimal eluent system. After checking up the TLC under UV, the best separation was obtained with a mixture of ethyl acetate: methanol: distillated water (8:1:1). Thus, this mobile phase was used for further experiments including bioautography and chromatographic separations.

The immersion bioautography procedure was performed on Silica gel TLC plates F_254_. Chromatograms were aseptically cut and placed in 9 cm × 9 cm sterile square Petri dishes and exposed to UV light for 30 min. The crude extract (1 mg/mL) was applied to the chromatograms and eluted with the previous mobile phase. The developed TLC plates were dried and coated with 15 mL of inoculated bacterial medium (10^6^ CFU/mL). After medium solidification, the Petri dishes were incubated at 37 °C for 24 h. Inhibitory zones were detected under UV light of 365 and 254 nm [24,25].

#### 2.5.2. Separation and Isolation of Pure Compounds

**Figure 1:**
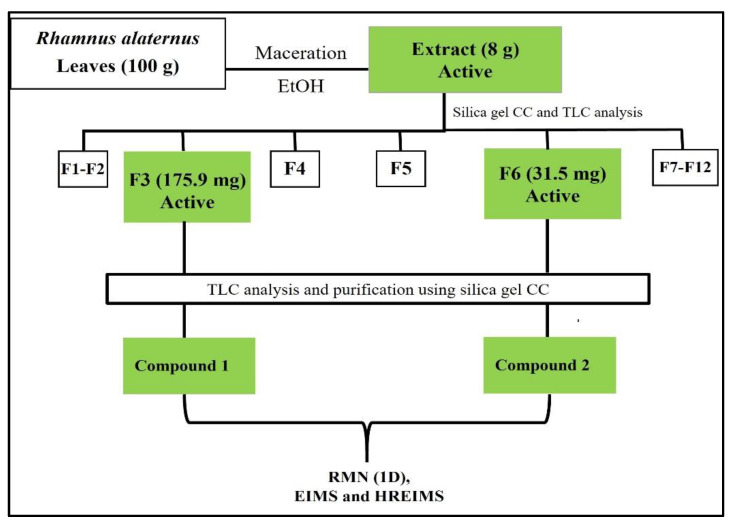
Flowchart of anti-staphylococcal bio-guided fractionation of *R. alaternus* leaves.

To isolate the active compounds detected by bioautography against the four *S. aureus* strains, 8 g of the crude extract was dissolved in ethanol and subjected to silica gel column chromatography eluted with ethyl acetate: methanol: distillated water (8:1:1). This afforded forty-six fractions combined into twelve fractions based on the TLC profile and coded from F1 to F12. According to the antibacterial assay, F3 (175.9 mg) and F6 (31.5 mg) were the most active and were further chromatographed on a silica column eluted with a mixture of dichloromethane/acetone (CH_2_Cl_2_/ Me_2_CO) of increasing polarity. As results, subfraction F3A yielded compound **1** (2.5 mg) while subfraction F6B yielded compound **2** (16.6 mg) [26]. The process of this bioguided fractionation is illustrated in Figure 1.

#### 2.5.3. Chemical Structures Characterization

The various chromatographic purification steps led to the isolation of two pure natural compounds. Samples of these both compounds were dissolved in appropriate deuterated solvents to be analyzed by ^1^H NMR, EIMS and HREIMS using Bruker Avance 600 spectrometer and an LCT Premier XE Micromass Electrospray Spectrometer. In addition, the obtained spectrums were compared with those previously reported in literature.

### 2.6. MIC Determination of Fractions and Pure Compounds

The MICs of samples obtained from fractionation were determined using the broth microdilution assay using modified 96-well plate following the recommendations published by CLSI for the effective assessment of the antimicrobial potency of natural products (Clinical and Laboratory Standards Institute 2009). In 96-well plates, a ½ dilution range of fractions (0.78125–500 µg/mL) and pure compounds (0.48827–250 µg/mL) was considered. The plates were then inoculated with 50 µL of the bacterial suspension adjusted to a density of 5 × 10^5^ UFC/mL. The growth control contained 50 μL of bacterial suspension and 50 μL of LB medium, while the negative control contained 100 µL of LB medium. The plates were incubated at 37 °C for 24 h. MIC values were determined after the addition of 10 µL of resazurin 0.015% (*w/v*) to each well and the plate was re-incubated at 37 °C for 2 h.

### 2.7. Cytotoxicity Assay

Cytotoxicity test was performed as described by Sifaoui et al. [27], different concentrations of compounds (**1**) were incubated with the J774A.1 murine macrophage cell line cultured in RPMI medium supplemented with 10% fetal bovine serum (10^5^ cells/mL) for 24 h at 37 °C in a 5% CO_2_ atmosphere. The viability of the macrophages was determined with the AlamarBlue reagent. Dose response curves were plotted and the CC_50_ was obtained. The analyses were performed in triplicate.

## 3. Results

### 3.1. Preliminary Antistaphylococcal Screening

In the present work, ethanolic extracts of fifteen plants used in traditional medicine for treatment of infectious diseases such as skin infections, in the Central North of Morocco [28], were evaluated. Leaves of *Ammi majus*, *Artemisia herba alba, Cistus salviifolius, Globularia alypum*, *Inula viscosa*, *Lavandula dentata*, *Nerium oleander*, and *Rhamnus alaternus*; aerial parts of *Crataegus oxyacantha* and *Urtica dioica*; roots of *Alkanna tintoria*, *Berberis hispanica*, *Ephedra altissima* and *Rubia tinctorum*; seeds of *Juniperus oxycedrus*. The anti-staphylococcal activity of the extracts was determined using the well-diffusion method against clinical isolate and reference strains of *S. aureus* with distinct characteristics and different antibiogram profiles. The diameters of the inhibitory zones were measured (in mm). The results obtained showed that among the fifteen ethanolic extracts tested, six extracts were active against the target strains, but with different inhibitory zones. Qualitatively, the ethanolic extracts of *B. hispanica* and *R. alaternus* showed the best activity against all strains, with a more remarkable susceptibility of *Sa* and MRSA (ATCC29213) compared to *Sa*PVL+ and MRSA348 (Table 1).

### 3.2. MIC Determination

The six active extracts were subjected to MIC determination using agar diffusion method. According to the results presented in Table 2, the extracts showed varying degrees of activity against the different strains of *S. aureus*. The extract of *R. alaternus* showed the best inhibitory effect with a MIC of 0.5 mg/mL against *Sa* and MRSA (ATCC29213), and 1.0 mg/mL against *Sa*PVL+ and MRSA348. The other extracts showed MICs between 4 and 16 mg/mL.

The serial dilution ranges from 16 to 0.25 mg/mL; the positive control (spots of 5 µL of the bacterial suspension on the agar medium) showed normal growth.

Based on the results of qualitative and quantitative tests, the best anti-staphylococcal effect was obtained by the extract of *R. alaternus*. Therefore, this plant was selected for further assays aimed at isolating and identifying the compounds responsible for this activity.

### 3.3. Bioguided Fractionation and Identification of Active Compounds

To isolate the compounds involved in the promising activity of *R. alaternus* against *S. aureus*, the ethanolic extract was subjected to a bioguided fractionation-alternating chemical and biological tests, followed by several separations and chromatographic purification steps. As a result, forty-six fractions were collected, combined according to their TLC profiles into twelve fractions (F1-F12) and tested against *S. aureus* strains. The MIC determination of the fractions obtained was performed using the microdilution method with resazurin as a staphylococcal viability indicator at a concentration ranging from 500 to 0.7812 µg/mL. The results obtained are presented in Table 3. As expected from the preliminary results of the bioautography which showed optimal zones of inhibition, fractions F3 (175.9 mg) and F6 (31.5 mg) were the most active with MICs between 31.5 and 200 µg/mL. *Sa*PVL+, MRSA348, and MRSA (ATCC29213) showed the same susceptibility level to both fractions, while *Sa* was the most susceptible to F3 with a MIC of 31.5 µg/mL, but the least susceptible to F6 with a MIC of 200 µg/mL.

Based on the results obtained, only the active fractions (F3 and F6) were selected for further purification using other silica gel chromatographic columns leading to the isolation of two pure natural compounds. The sub-fraction of F3 gave compound (**1**) (2.5 mg), and the purification of F6 gave compound (**2**) (16.6 mg).

**Figure 2:**
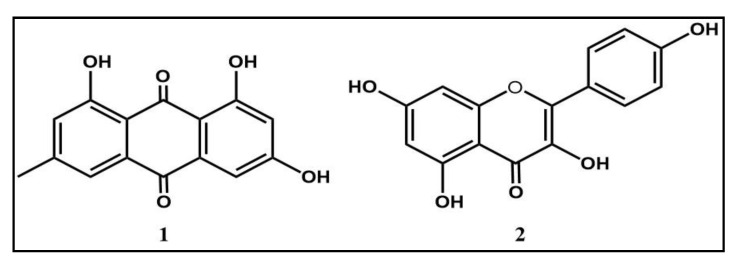
Chemical structures of emodin (**1**) and kaempferol (**2**) isolated from the leaves of *R. alaternus*.

NMR analyses (^1^H and ^13^C) of these compounds led to the identification of emodin (**1**) kaempferol (**2**) (Figure 2). The spectroscopic data from the NMR spectra (Figures S3 and S4) corroborate those previously reported in the literature [29,30]. In addition, mass spectrometry analyses were performed to confirm the structures obtained (Figures S5–S8).

After confirmation of the chemical structures of the two compounds, the determination of MICs and BMCs was performed by the microdilution method still using resazurin as an indicator of staphylococcal viability at a concentration range from 250–0.4882 µg/mL. In addition, the cytotoxic profile of the active compound was evaluated using AlamarBlue as an indicator of murine macrophage viability (Table 4).

Based on Table 4, we can note that emodin strongly inhibited the growth of the tested strains with a MIC of 15.63 µg/mL against *Sa*PVL+, MRSA348, and MRSA (ATCC29213), and a much lower MIC against *Sa* (1.95 µg/mL). However, kaempferol was not active at the concentration tested (>250 µg/mL).

### 3.4. Cytotoxicity Assay

The in vitro cytotoxicity of emodin as the active compound was evaluated by calculating the percentage of living macrophage cells regarding different concentrations. Subsequently, the curve obtained allows the calculation of CC_50_s corresponding to the concentration of the compound for which there is a 50% reduction in cell viability compared to untreated macrophages. The determination of the CC_50_s allows the calculation of the selectivity index. The results showed that emodin was not toxic on the J774A.1 macrophage cell line (CC_50_ > 100 µg/mL).

## 4. Discussion

*S. aureus* is one of the pathogens involved in a wide clinical spectrum. The emergence of multidrug-resistant and toxigenic strains is a serious public health problem. A multitude of studies have confirmed that the rate of infections worldwide is progressively increasing with the rise of drug resistance and that clinical anti-infective treatment of resistant strains has become more difficult [31,32] especially when the infections are caused by methicillin-resistant and/or Panton-Valentine leukocidin positive strains. Indeed, MRSA strains were once largely confined to hospitals, health care services, and patients attending these facilities, known as HA-MRSA. Unfortunately, new strains of MRSA, often referred to as community-associated MRSA (CA-MRSA) strains, appear to be rapidly disseminated in the population and infect people with and without hospital exposure [33]. These CA-MRSA strains participate in widespread severe MRSA infections [34,35].

Therefore, a special interest was taken in the search for new compounds of natural origin. In the present work, screening tests on different strains of *S. aureus* showed that among the fifteen ethanolic extracts prepared from various plant species, the ethanolic extract of *R. alaternus* was the most active against the four target strains of *S. aureus*. We suppose that these results could be explained, on the one hand, by the differences in the permeability of the cell walls to secondary metabolites, and on the other hand, by the differences in the chemical composition of the plants leading to a different mechanism of action. In other words, the extract may contain one or more compounds that are specifically more effective against various strains of *S. aureus* characterized by different pathogenic and resistant profiles. Despite the antibacterial potential of *R. alaternus* in the present study, very limited data are available in the literature regarding its antimicrobial effect, especially the anti-staphylococcal activity. It was reported that evaluation of the antibacterial activity of nine extracts and fractions of the leaves of *R. alaternus* showed that this species exhibits a broad spectrum of antibacterial activity against a wide list of pathogens including a reference strain of *S. aureus* (ATCC25923). Only ethyl acetate extracts, total flavonoid oligomers, and the ethyl acetate fraction obtained from freeze-drying of the aqueous extract showed antibiotic activity against *S. aureus* with MIC values ranging from 70 to 150 µg/mL; phytochemical screening of these extracts revealed their richness in flavonoids and phenolic compounds [36]. Kosalec et al. have also evaluated the antimicrobial potential of methanolic bark extracts of four species of *Rhamnus* genus against several bacteria, dermatophytes, and yeasts. They have demonstrated that the extract of *R. alaternus* inhibited the growth of *S. aureus* but with a MIC of 2.5 mg/mL and the analysis of the anthraquinone profile revealed the presence of chrysophanol as the main compound, emodin and physion [37]. Although in these studies the leaves and bark of *R. alaternus* inhibited the growth of *S. aureus*, in both studies the characterization and purification of the bioactive compounds responsible for the activity was not completed and the antibacterial activity was only tested on reference strains which do not really reflect the reality of clinical strains.

In contrast to antibacterial activities, the literature has focused much more on other previously documented pharmacological properties of *R. alaternus*. These studies have allowed the characterization of a large list of bioactive compounds but especially antioxidants. Purification procedures have highlighted the presence of flavonols such as kaempferol, rhamnetin and rhamnocitrin [16], anthocyanins [17] and anthraquinones such as emodin [18,19]. The identification of the compounds responsible for the anti-staphylococcal activity of *R. alaternus* in this work was performed by a bio-guided fractionation, spectroscopic and spectrometric analysis which confirms the abundance of anthraquinones and flavonoids. Our purification process identified emodin belonging to the anthraquinone family and kaempferol to the group of flavonoids, which agree with the literature. Furthermore, we have shown that emodin is the compound responsible for the antibacterial potential of *R. alaternus* showing a promising activity (MIC up to 1.95 µg/mL) with a non-toxic concentration toward murine macrophages.

Indeed, emodin is a secondary metabolite with a wide range of biological properties in vitro and in vivo: anticancer, antioxidant, anti-inflammatory, antiviral, antiallergic, antibacterial, etc., [38]. Although our study is the first to report the antibacterial effect of emodin isolated from *R. alaternus*, this compound is well-known for its anti-staphylococcal potential when isolated from other plants. Basu et al. have shown that emodin isolated from *Ventilago madraspatana* bark was active against *S. aureus* with an MIC of 90 µg/mL higher than the value reported in our study [39]. Chukwujekwu et al. have reported that emodin isolated from the roots of *Cassia occidentalis* is the responsible compound for the antimicrobial activity of the ethanolic extract especially against *S. aureus* with a MIC of 3.9 µg/mL highlighting a more interesting effect than the reference antibiotic, neomycin [40]. On the other hand, it was shown that emodin purified from the roots and rhizomes of *Rheum officinale* affected the thermogenic curves of *S. aureus* growth by improving the antimicrobial effect [41]. Similar effects were noted against MRSA and MSSA. Cao et al. have identified emodin as the active compound in *Polygoni cuspidati* extract. Among the seven major compounds purified, only emodin showed significant activity against a clinical strain of MRSA while causing morphological alterations of the cell wall [42]. Ji et al. have conducted a proteomic study to compare the effect of emodin on MRSA and MSSA strains. They have demonstrated that after treatment of both strains with emodin, protein expression levels were altered by multiple similar mechanisms of action in MRSA than in MSSA namely induction of pyruvate pathway imbalance, inhibition of protein synthesis, and suppression of DNA synthesis, in addition to membrane alterations [43]. In fact, these mechanisms of the action exerted by emodin have been widely confirmed in the literature [44,45,46].

By cross-analysis of the biological and phytochemical data, we can hypothesize the chemical nature of the bioactive compound in the present study, the activity and selectivity of emodin toward *S. aureus* strains may be related, for example, to functional groups on the phenyl ring of the compound. These findings were confirmed by the researchers. A structure–activity relationship (SAR) study indicated that the hydroxyl groups and the methyl group in the emodin backbone were crucial for anti-MRSA activity and the presence of an iodine atom or an ethylamine group on the aromatic ring improved the activity with higher selectivity index [47]. Furthermore, another study showed that the structure of emodin was strongly involved in the antimicrobial activity. The substitution at position 2 of emodin was detrimental to the antibacterial activity, while the unsaturation of the substitute was important for the activity. Thus, increasing the aliphatic chain length of the methoxyl group at position 3 increases the lipophilicity of the compound, which increases its ability to integrate the bacterial cell wall, which becomes permeable [48].

Nevertheless, emodin’s low solubility, poor bioavailability, and oral absorption limit its development into a pharmaceutical product. Several researchers have conducted in vivo trials on the pharmacokinetics of emodin in order to improve its solubility and oral bioavailability by following different approaches such as inhibition of crystallization, glucuronoconjugation, and addition of solubility enhancers such as cyclodextrin A and nanoparticles or combination of emodin with other molecules such as piperine and antibiotics [49,50,51,52,53,54].

In the present work, we have also shown that kaempferol was not active at the tested concentration, which corroborates the study reported by Falcão-Silva et al. where they have indicated that kaempferol glycoside isolated from *Herissantia tiubae* did not show relevant activity against *S. aureus* (MIC = 256 μg/mL), but rather this compound modulated the activity of antibiotics, which in combination with antibiotics, reduced MIC values by enhancing the putative efflux pump inhibitory effect in the bacteria [55].

Another important point to discuss in the present work is the evaluation of different strains of *S. aureus*. In fact, we cannot say that methicillin-resistant or multidrug-resistant strains are the only ones involved in invasive infections, but rather they limit the choice of antibiotic therapy. In this context, several studies have compared the involvement of methicillin-susceptible strains (MSSA) and MRSA in different infections. Ding et al. have evaluated whether there was a difference between breast abscesses caused by MRSA and MSSA. They have noted a dominance of MSSA isolates (N = 132) over MRSA (N = 39), but there was no significant difference in antibiotic use rate, cure rate, and median number of aspirations performed to cure [56]. However, in a prospective study, it was noted that MSSA caused 79% of infections, while MRSA caused only 21% concluding that during the study period MSSA was the main cause of invasive *S. aureus* infections but the classical virulence toxin PVL was rare in these isolates [57]. Furthermore, Sapri et al. have demonstrated that the most virulent genes, such as PVL, were more frequently found in MSSA than in MRSA. Taken together, these data with specific *S. aureus* strains, the authors have suspected an inverse relationship between resistance and virulence in *S. aureus* [58]. In other words, multidrug-resistant strains tend to harbor fewer virulent genes, while MSSA strains are more virulent but susceptible to antibiotics. This hypothesis was previously confirmed by Cameron et al., exploring available clinical and experimental data, they have shown that many major steps in the evolution of resistance in *S. aureus* are accompanied by virulence alterations [59]. However, other researchers have reported that increased virulence and the development of antibiotic resistance often occur almost simultaneously, but in complex genetic relationships [60].

## 5. Conclusions

Among the 15 plants selected for the in vitro inhibition of MRSA and MSSA-PVL, *R. alaternus* was the most active. The bioguided fractionation of the extract of this plant yielded emodin as the active compound without inducing toxicity toward murine macrophages. However, further in vivo pharmacological research will be required to fully understand the pharmacokinetics of the active compound, to improve its solubility and to evaluate a possible synergistic interaction with other compounds leading to an effective anti-staphylococcal formulation.

## 6. Patent

Results about fraction 5 (Data not shown) have been subjected to a patent.

## Figures and Tables

**Table 1:** Inhibition zones of ethanolic extracts of the fifteen plants against *S. aureus* strains.

Ethanolic Extracts		*S. aureus* Strains		
	*Sa*PVL+	MRSA348	*Sa*	MRSA (ATCC29213)
*R. alaternus*	14 ± 1.0	15 ± 1.0	25.5 ± 1.5	25 ± 1.0
*I. viscosa*	12 ± 1.0	12 ± 0.81	13.66 ± 1.88	13 ± 0.81
*C. oxyacantha*	11± 0.5	14 ± 0.5	12.33 ± 1.88	11.66 ± 1.24
*R. tinctorum*	10 ± 0.5	20 ± 0.5	10.33 ± 0.47	12.5 ± 0.5
*A. herba alba*	13 ± 1.88	10 ± 0.0	14.5 ± 2.12	13 ± 0.7
*B. hispanica*	15 ± 0.0	20 ± 1.0	26 ± 1.0	24 ± 1.0
*E. altissima*	NA	NA	NA	NA
*L. dentata*	NA	NA	NA	NA
*C. salviifolius*	NA	NA	NA	NA
*N. oleander*	NA	NA	NA	NA
*A. tinctoria*	NA	NA	NA	NA
*J. oxycedrus*	NA	NA	NA	NA
*U. dioica*	NA	NA	NA	NA
*G. alypum*	NA	NA	NA	NA
*A. majus*	NA	NA	NA	NA

Inhibition zones (mm) including the disc diameter 6 mm; NA: not active.

**Table 2:** Minimum inhibitory concentrations of ethanolic extracts of the six plants selected using the qualitative test against *S. aureus* strains.

Ethanolic Extracts		MIC (mg/mL)		
	*Sa*PVL+	MRSA348	*Sa*	MRSA (ATCC 29213)
*A. herba alba*	>16	16	8	8
*B. hispanica*	>16	16	16	16
*C. oxyacantha*	16	16	16	16
*I. viscosa*	8	4	4	4
*R. alaternus*	1.0	1.0	0.5	0.5
*R. tinctorum*	>16	8	16	16

**Table 3:** Minimum inhibitory concentrations of *R. alaternus* fractions against the four strains of *S. aureus*.

Strains	*Sa*PVL+	MRSA348	*Sa*	MRSA (ATCC 29213)
*R. alaternus*		MIC (µg/mL)		
Crude extract	1000	1000	500	500
F1-F2	NA	NA	NA	NA
F3	125	125	31,5	125
F4	NA	NA	NA	NA
F5	DN	DN	DN	DN
F6	50	50	200	50
F7-F12	>1000	>1000	>1000	>1000
Control +	NG	NG	NG	NG
Control −	NG	NG	NG	NG

Control +: strain and LB medium; control −: strain and 2% DMSO; NG: normal growth. DN: data not shown.

**Table 4:** Minimum inhibitory concentrations of pure compounds isolated from *R. alaternus* fractions against the four strains of *S. aureus* cytotoxicity test and selectivity index against murine macrophages.

Compound/Strain	*Sa*PVL+		MRSA348		*Sa*		MRSA (ATCC 29213)		Murin Macrophages
	MIC (µg/mL)	SI	MIC (µg/mL)	SI	MIC (µg/mL)	SI	MIC (µg/mL)	SI	CC_50_ (µg/mL)
Emodin	15.63	>6.4	15.63	>6.4	1.95	>51.28	15.63	>6.4	>100
Kaempferol	>250	-	>250	-	>250	-	>250	-	-
Control +	NG		NG		NG		NG		
Control −	NG		NG		NG		NG		

Control +: strain and LB medium; control −: strain and 2% DMSO; NG: normal growth. BMC of emodin >250 µg/mL. MIC: minimum inhibitory concentration. CC_50_: cytotoxic concentration that reduces viability of murine macrophages by 50%. SI: CC_50_/MIC.

## Data Availability

Data are available within the text and from corresponding authors.

## Supplementary Materials

The following are available online. Table S1: The susceptibility test of *S. aureus* strains (*Sa* and MRSA (ATCC 29213)); Table S2: The susceptibility test of *S. aureus* strains (MRSA348 et *Sa*PVL+); Figure S3: ^1^H NMR spectrum of emodin (600 MHz, CDCl3); Figure S4: ^1^H NMR spectrum of kaempferol (600 MHz, C3H6O); Figure S5: EIMS spectrum of emodin; Figure S6: EIMS spectrum of kaempferol; Figure S7: HREIMS spectrum of emodin; Figure S8: HREIMS spectrum of kaempferol.
