# Supplementary material for: Bioguided Isolation of Active Compounds from Rhamnus alaternus against Methicillin-Resistant Staphylococcus aureus (MRSA) and Panton-Valentine Leucocidin Positive Strains (MSSA-PVL)
Source: Molecules. 2021 Jul 19;26(14):4352. doi: 10.3390/molecules26144352 (PMC8306708; doi:10.3390/molecules26144352)
Supplement: Supplementary file 1 [file molecules-26-04352-s001.zip › molecules-1285630-supplementary.pdf]

Bioguided isolation of active compounds from *Rhamnus alaternus* against Methicillin Resistant *Staphylococcus aureus* (MRSA) and Panton-Valentine leucocidin positive strains (MSSA-PVL)

Ikrame Zeouk [1,2,*], Wessal Ouedrhiri[3], Ines Sifaoui [1,4,5], Isabel L. Bazzocchi [6],

José E. Piñero [1,4,5,*], Ignacio A. Jiménez [6], Jacob Lorenzo-Morales [1,4,5,*], Khadija Bekhti [2]

[1] Instituto Universitario De Enfermedades Tropicales y Salud Pública de Canarias, Universidad de La Laguna, Avda. Astrofísico Fco. Sánchez, S/N, La Laguna, Tenerife, Islas Canarias 38203, Spain

[2] Departement of Biology, Sidi Mohamed Ben Abdellah University, Faculty of Sciences and Techniques, Laboratory of Microbial Biotechnology and Bioactive Molecules, PB 2202, Fez, Morocco

[3] Department of Chemistry, Sidi Mohamed Ben Abdellah University, Faculty of Science, Laboratory of Engineering, Electrochemistry, Modeling and Environment

[4] Departamento de Obstetricia, Ginecología, Pediatría, Medicina Preventiva y Salud Pública, Toxicología, Medicina Legal y Forense y Parasitología, Universidad De La Laguna, La Laguna, Tenerife, Islas Canarias 38203, Spain

[5] Red de Investigación Colaborativa en Enfermedades Tropicales (RICET)

[6] Instituto Universitario de Bio-Orgánica Antonio González, Departamento de Química Orgánica, Universidad de La Laguna, Avenida Astrofísico Francisco Sánchez 2, 38206 La Laguna, Tenerife, Spain

***Corresponding authors**

Corresponding author details

Ikrame ZEOUK (IZ), Instituto Universitario De Enfermedades Tropicales y Salud Pública de Canarias, Universidad de La Laguna, Avda. Astrofísico Fco. Sánchez, S/N, La Laguna, Tenerife, Islas Canarias 38203, Spain
E-mail: ikramezeouk20@gmail.com
Phone: 212621290377

Jacob LORENZO-MORALES (JLM), Instituto Universitario De Enfermedades Tropicales y Salud Pública de Canarias, Universidad de La Laguna, Avda. Astrofísico Fco. Sánchez, S/N, La Laguna, Tenerife, Islas Canarias 38203, Spain
E-mail: jmlorenz@ull.edu.es
Phone: +34922318402

José E. PIÑERO (JEP), Instituto Universitario De Enfermedades Tropicales y Salud Pública de Canarias, Universidad de La Laguna, Avda. Astrofísico Fco. Sánchez, S/N, La Laguna, Tenerife, Islas Canarias 38203, Spain

E-mail: jpinero@ull.edu.es
Phone: +34922316502

S1. **The susceptibility test of *S. aureus* strains (*Sa* and MRSA (ATCC 29213))**

S2. **The susceptibility test of *S. aureus* strains (MRSA348 et *Sa*PVL+)**

S3. **^{1}H NMR spectrum of emodin (600 MHz, $CDCl_3$)**

S4. **^{1}H NMR spectrum of kaempferol (600 MHz, C_3H_6O)**

S5. **EIMS spectrum of emodin**

S6. **EIMS spectrum of kaempferol**

S7. **HREIMS spectrum of emodin**

S8. **HREIMS spectrum of kaempferol**

S1. The susceptibility test of *S. aureus* strains (*Sa* and MRSA (ATCC 29213)).

Antibiotics family	Antibiotics	Dose par disc (µg)	*S. aureus* strains	
			Sa	MRSA (ATCC 29213)
Penicillins	Penicillin	10 units	Resistant	Resistant
	Ampicillin	10	Resistant	Resistant
	Amoxicillin	20	Resistant	Resistant
	Oxacillin	1	Resistant	Resistant
	Methicillin	5	Resistant	Resistant
Penicillin combination	Clavulanic acid + amoxicillin	10/20	Resistant	Resistant
Cephalosporines	Ceftriaxone	30	Resistant	Resistant
	Ceftazidime	30	Resistant	Resistant
Glycopeptides	Vancomycin	30	Susceptible	Susceptible
	Teicoplanine	30	Susceptible	Susceptible
Macrolides	Erythromycin	15	Resistant	Resistant
	Spiramycin	15	Resistant	Resistant
Tetracyclines	Tetracycline	30	Susceptible	Susceptible
Polypeptides	Colistin	10	Resistant	Resistant
Others	fusidic acid	10	Resistant	Resistant
	Pristinamycin	10	Susceptible	Susceptible

S2. The susceptibility test of *S. aureus* strains (MRSA348 et *Sa*PVL+).

Antibiotics family	Antibiotics	*S. aureus* strains	
		MRSA348	*Sa*PVL+
Aminoglycosides	Gentamicin	Resistant	Susceptible
Aminosides	Tobramycin	Resistant	Susceptible
Penicillins	Penicillin	Resistant	Resistant
	Methicillin	Resistant	Susceptible
Cephamycins	Cefoxitin	Resistant	Susceptible
Macrolides	Erythromycin	Resistant	Susceptible
Tetracyclines	Tetracycline	Resistant	Susceptible
Quinolones	Norfloxacin	Resistant	Susceptible
Rifamycins	Rifampicin	Susceptible	Susceptible
Others	Fusidic acid	Resistant	Susceptible
	Clindamycin	Susceptible	Susceptible
	Trimethoprim/Sulfamethoxazole	Resistant	Susceptible

MRSA348: absence of PVL toxin; *Sa*PVL+: produce PVL toxin.

S3. ^{1}**H NMR spectrum of emodin (600 MHz,** $CDCl_3$**).**

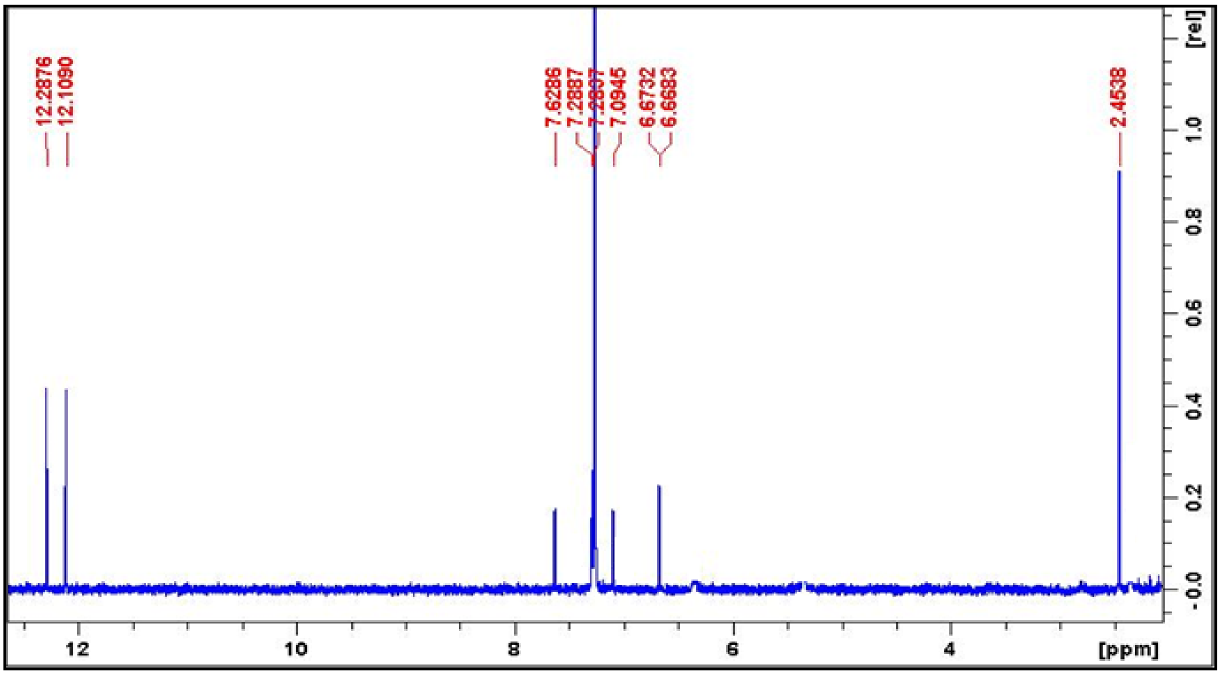

S4. ^{1}**H NMR spectrum of kaempferol (600 MHz,** C_3H_6O**)**

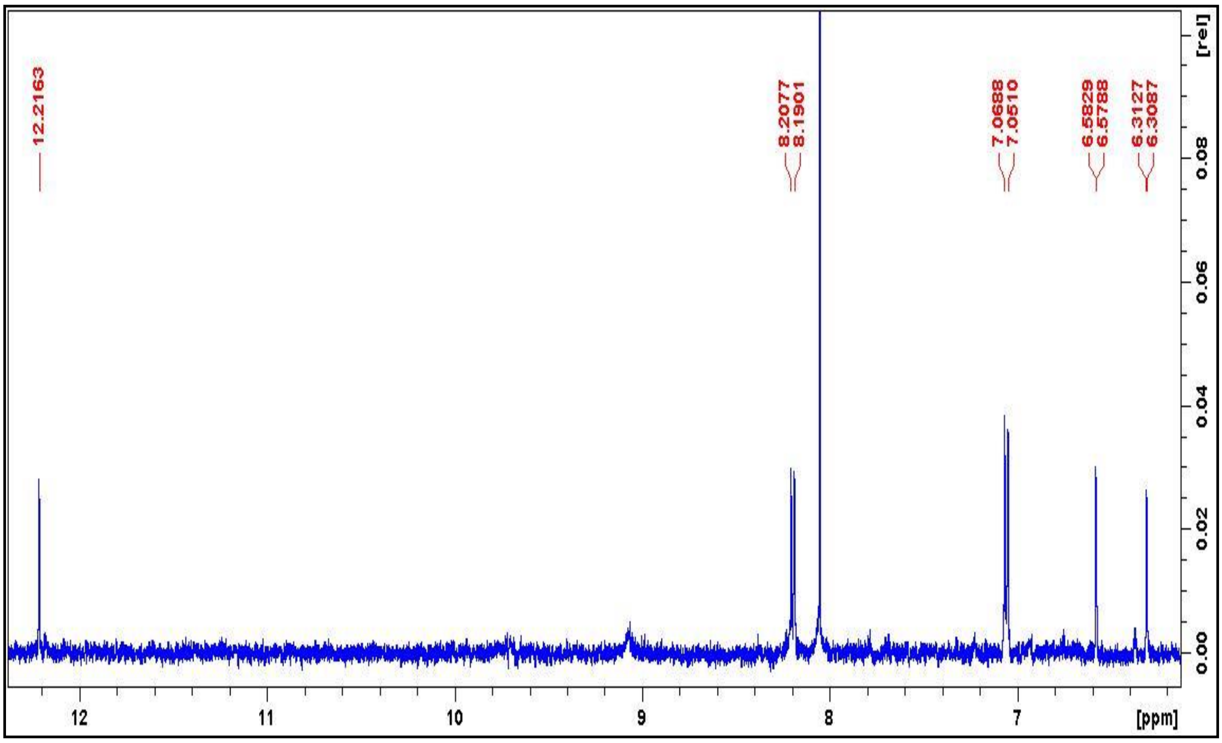

S5. EIMS spectrum of emodin

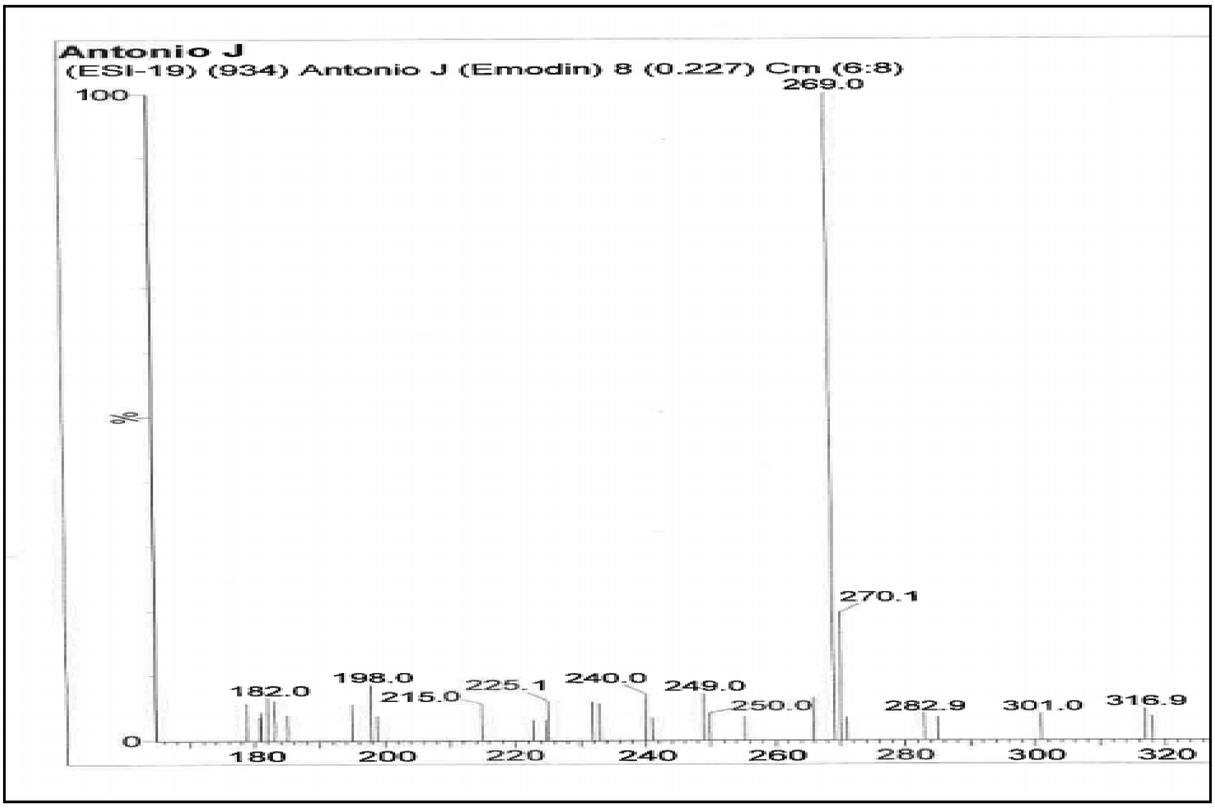

S6. EIMS spectrum of kaempferol

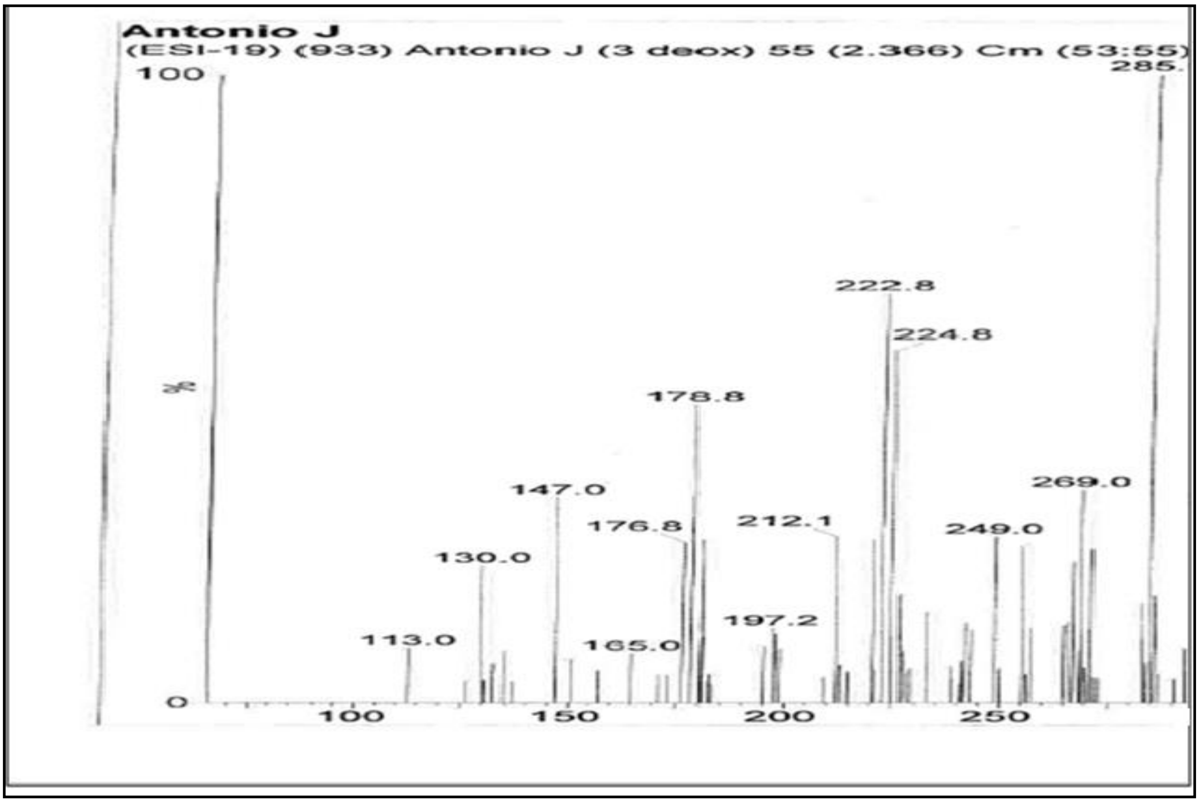

S7. HREIMS spectrum of emodin

Elemental Composition Report **Page 1**

Tolerance = 5.0 PPM / DBE: min = -3.0, max = 120.0
Element prediction: Off
Number of isotope peaks used for i-FIT = 3

Monoisotopic Mass, Even Electron Ions
115 formula(e) evaluated with 1 results within limits (all results (up to 1000) for each mass)
Elements Used:
C: 0-60 H: 0-60 O: 0-6 Na: 0-2 I: 0-2

Antonio J
(ESI-19) (934) Antonio J (Emodin) 8 (0.227) 2: TOF MS ES- 7.99e+003

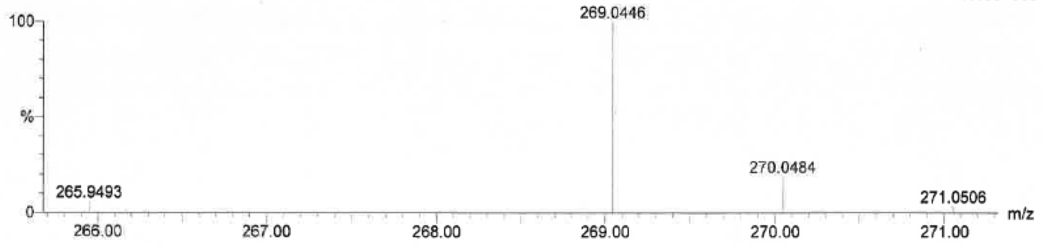

Minimum:	20.00				-3.0			
Maximum:	100.00		5.0	5.0	120.0			
Mass	RA	Calc. Mass	mDa	PPM	DBE	i-FIT	i-FIT (Norm)	Formula
269.0446	100.00	269.0450	-0.4	-1.5	11.5	9.8	0.0	C15 H9 O5

S8. HREIMS spectrum of kaempferol

Elemental Composition Report **Page 1**

Tolerance = 5.0 PPM / DBE: min = -3.0, max = 120.0
Element prediction: Off
Number of isotope peaks used for i-FIT = 3

Monoisotopic Mass, Even Electron Ions
125 formula(e) evaluated with 1 results within limits (all results (up to 1000) for each mass)
Elements Used:
C: 0-60 H: 0-60 O: 0-6 Na: 0-2 I: 0-2

Antonio J
(ESI-19) (933) Antonio J (3 deox) 44 (1.878) 1: TOF MS ES- 1.00e+003

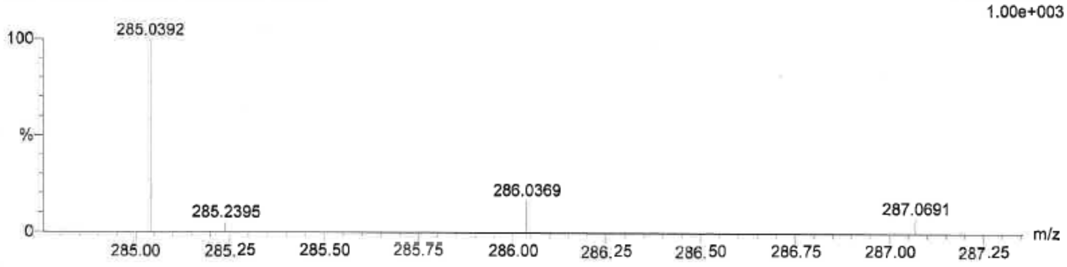

Minimum:	20.00				-3.0			
Maximum:	100.00		5.0	5.0	120.0			
Mass	RA	Calc. Mass	mDa	PPM	DBE	i-FIT	i-FIT (Norm)	Formula
285.0392	100.00	285.0399	-0.7	-2.5	11.5	26.3	0.0	C15 H9 O6
